# Dynamic probe selection for studying microbial transcriptome with high-density genomic tiling microarrays

**DOI:** 10.1186/1471-2105-11-82

**Published:** 2010-02-09

**Authors:** Hedda Høvik, Tsute Chen

**Affiliations:** 1 Department of Oral Biology, Faculty of Dentistry, University of Oslo, Oslo, Norway; 2 Department of Molecular Genetics, The Forsyth Institute, Boston, MA, USA

## Abstract

**Background:**

Current commercial high-density oligonucleotide microarrays can hold millions of probe spots on a single microscopic glass slide and are ideal for studying the transcriptome of microbial genomes using a tiling probe design. This paper describes a comprehensive computational pipeline implemented specifically for designing tiling probe sets to study microbial transcriptome profiles.

**Results:**

The pipeline identifies every possible probe sequence from both forward and reverse-complement strands of all DNA sequences in the target genome including circular or linear chromosomes and plasmids. Final probe sequence lengths are adjusted based on the maximal oligonucleotide synthesis cycles and best isothermality allowed. Optimal probes are then selected in two stages - sequential and gap-filling. In the sequential stage, probes are selected from sequence windows tiled alongside the genome. In the gap-filling stage, additional probes are selected from the largest gaps between adjacent probes that have already been selected, until a predefined number of probes is reached. Selection of the highest quality probe within each window and gap is based on five criteria: sequence uniqueness, probe self-annealing, melting temperature, oligonucleotide length, and probe position.

**Conclusions:**

The probe selection pipeline evaluates global and local probe sequence properties and selects a set of probes dynamically and evenly distributed along the target genome. Unique to other similar methods, an exact number of non-redundant probes can be designed to utilize all the available probe spots on any chosen microarray platform. The pipeline can be applied to microbial genomes when designing high-density tiling arrays for comparative genomics, ChIP chip, gene expression and comprehensive transcriptome studies.

## Background

With the advancement of microarray technology, commercial high-density oligonucleotide microarrays can hold millions of probes on a single microscopic glass slide. Microarray technology can currently print up to five million features per microarray slide using the *in situ *oligo synthesis method [1]. Maskless array synthesis (MAS) technology [2] makes it possible to synthesize oligonucleotides of any sequence. This great capacity can be used for designing probe sequences covering an entire small genome or chromosome or a continuous portion of larger genomes. Probes can be designed to closely 'tile' along the genomic sequence so that any two neighboring probes are immediately adjacent to or overlapping each other.

The hybridization signals detected by the tiling designed probes can be plotted on the genomic coordinates to form a comprehensive transcription profile. The profile reveals all the RNA transcripts in the cells comprising both protein and non-protein coding RNAs including those that are not predicted by computational annotation. Genomic tiling arrays are thus useful in both confirming the computer-predicted genes and discovering novel RNAs. They have been used to study whole genome or chromosome transcription for various organisms including humans [3-6]. For small genomes such as those of prokaryotes, the capacity of the high-density tiling arrays often allows for studies of the comprehensive transcriptome [7-9].

Compared to eukaryotes, prokaryotes (including both Archaea and Bacteria) generally have smaller genomes and the genes are more densely packed. The majority of prokaryotic genomes are smaller than 6 Mbp [10] with an average non-protein coding portion of only 12% [11]. Higher eukaryotes have a much larger portion non-protein to protein coding sequences, e.g. the human genome with only ~2.1% exonic DNA [11]. Most of the eukaryotic non-protein coding sequences are low complexity sequences, such as repetitive elements [12]. Interestingly many of the non-protein coding sequences are functionally active and have been found to play important regulatory roles both in eukaryotes [13,14] and prokaryotes [15]. Thus in terms of studying transcriptome profiles, these regions should not be masked or neglected when designing probes for genomic tiling arrays as this may result in overlooking novel and active transcripts with important functions.

The most straightforward method for designing genomic tiling arrays simply selects a probe at fixed intervals across the entire genome without considering any factors affecting the hybridization. This is called naïve tiling and has been used in transcriptome studies for organisms such as *Escherichia coli *[7] and *Thalassiosira pseudonana *[16]. More sophisticated tiling design algorithms were later developed and have been reviewed by Schliep and Krause [17] and by Lemoine *et al. *[18]. In general, probe sets can be selected by generating a tile path [12,17] or by selecting probes from either fixed [19] or more dynamic selection windows [20]. Gräf *et al*. [19] used an algorithm calculating and sorting all potential probes according to a uniqueness score. Within a defined selection window the probe candidate with the highest uniqueness score is identified. However, Gräf's algorithm may reject all probe candidates in a window leaving an area on the genome without complete probe coverage. Similarly Lipson *et al. *[20] used an approach to select the best probes of high local quality within predefined limits for both quality and distance. More recently Schliep and Krause [17] reported an optimal tile path algorithm for designing tiling probe sets. This algorithm was designed in particular for selecting non-overlapping probes with dynamic distances between adjacent probes to allow highest quality of probe sequences. Another approach for non-equidistant tiling used by Thomassen *et al*. [21], divided the genomic sequence into regions according to different levels of interest (e.g. coding and non-coding regions).

The aim of this paper is to describe a probe selection computational pipeline for designing tiling probe sets, originally developed to study transcriptome profiles of prokaryotes. All possible probe sequences of the target genome are evaluated and optimal probes are then dynamically selected in two stages - sequential and gap-filling. In the first sequential stage, probes are selected from fixed sized sequence windows tiled alongside the genome. By gap-filling additional probes are selected from the largest gaps between existing already selected probes. Compared to eukaryotic genomes, which tend to have larger areas of repetitive sequences giving uninformative hybridization signals due to cross-hybridization, the gap-filling increases the probe coverage generating valuable information when applied to the smaller and compact microbial genomes. Combining the two different methods provides the opportunity to emphasize either sequential or gap selection. The gap-filling also allows for the selection of additional probes until a desired number of total probes is reached, making it possible to utilize every probe spot on any microarray platform. Unlike most probe design applications, no cut-off limits are applied to ensure that one best probe representative always is selected from every window, and consequently all areas of the genome are covered. The uniqueness of the pipeline is the generation of a probe set holding an exact number of non-redundant oligonucleotide sequences that are evenly tiled along the target microbial genome. Overall, this pipeline provides a fine balance between probe coverage and quality for designing genomic tiling array probe sets to study microbial transcriptome profiles.

## Implementation

### Algorithm overview

**Figure 1:**
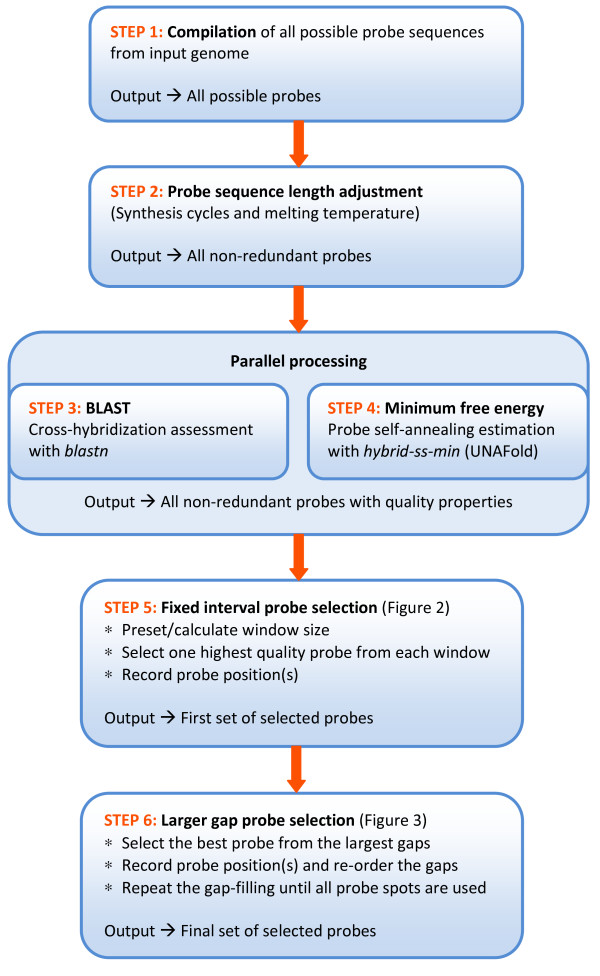
**Overview of the tiling array probe design pipeline**.

The algorithm described is a pipeline of several computer scripts specifically developed for selecting a set of tiling probes from the genomic sequence. The pipeline input is the whole genomic sequence of the target genome. The final output is a probe set of non-redundant oligonucleotide sequences dynamically and evenly distributed across the entire input sequence. The pipeline consists of six major steps (Figure 1), described as follows:

### Step 1: Compilation of all possible probe sequences from a genome

As many genomes contain multiple circular and/or linear sequences, all genomic molecules of the target organism are included in the probe sequence compilation. For circular sequences, a portion of the sequence from the first base of the 5'-end, with a length equal to that of the probe minus one, is appended to the 3'-end of the sequence so that probe sequences crossing the start-end junction are accounted for. All the genomic sequences are concatenated to form a single pseudo-molecule with spacer sequences. The script will then compile all the possible probe sequences of desired length starting from the first base of the pseudo-molecule moving forward one base at a time. The reverse-complement sequence is saved at the same time. Sequences containing any non-ATCG code are omitted which makes it possible to process both pre-masked and spacer sequences.

### Step 2: Adjustment of probe sequence length by synthesis cycles and melting temperature

The sequence length of each probe is adjusted according to two parameters:

#### Oligonucleotide synthesis cycles

Our custom microarrays were printed by Roche NimbleGen, Inc. (Madison, WI, USA) using the maskless array synthesizer (MAS) technology with a preset maximal number of synthesis cycles. The number of synthesis cycles for each probe sequence is calculated and when needed the probe is shortened from the 3'-end of the sequence until the number of synthesis cycles is smaller or equal to the maximal limit.

#### Melting temperature

The melting temperature (Tm) of a probe sequence affects the hybridization efficiency with its complementary target [22]. An isothermal probe design facilitates similar binding kinetics between probes and targets in the hybridization solution and thereby comparable signal intensities. Adjustment of probe sequence length is applied to obtain the best isothemality possible. Due to multiple Tm calculations for various lengths of every single probe sequence we implemented a less computational time consuming salt adjusted method, even though the nearest-neighbor method is reported to be more accurate [23]. For longer probe sequences (>50 nucleotides) a salt and optional formamide adjusted equation is used [24]:

A slightly different salt adjusted equation is applied for shorter probe sequences [23-25]:

In both formulas, ***N ***is the total number of nucleotides in the probe sequence, ***G ***and ***C ***the number of respective nucleotides, ***[Na^+^] ***the monovalent salt molar concentration, and ***F ***the formamide percent concentration. Melting temperatures of all probe sequences are calculated and the median Tm value recorded. Probe sequences with a Tm different from the median Tm are truncated one base at the 3'-end and the Tm is then recalculated. Truncation and recalculation is repeated until the Tm is equal to the median Tm or a preset minimal probe sequence length is reached and the probe sequence with Tm closest to the median Tm will then be used.

The output of this step is the comprehensive collection of all possible non-redundant probe sequences including the genomic position. For repeated sequences multiple positions are recorded.

### Step 3: Assessment of cross-hybridization

Sequence complementarities between a probe and any unintended target (non-target) can cause cross-hybridization and thereby generate false positive signals that interfere with the identification of true ones. Every probe sequence generated in Step 2 is subjected to a BLAST [26] search against the pseudo-molecule made in Step 1. Cross-hybridization is evaluated by observing the percentage of sequence identity (i.e., complementarities) between the probe sequence and non-target sequences and by the length of continuous complementary sequence stretch with non-target sequences, referred to as identity stretch. Many studies have used sequence percentage identity as either the single parameter or in combination with identity stretch to evaluate cross-hybridization [27-32]. Based on results from these studies we find it feasible to sort the probe sequences according to levels of expected cross-hybridization potential and the default level limits were selected as follows, where PI is the percent identity and IS the identity stretch (as the percentage of probe sequence length):

Level 1 - PI < 50%

Level 2 - 50% ≤ PI < 75% and IS < 30%

Level 3 - 75% ≤ PI < 90% and 30% ≤ IS < 40%

Level 4 - PI ≥ 90% and IS ≥ 40%

Level 1 and 2 reflect virtually no cross-hybridization potential and the predefined default limits were based on results reported by Kane *et al. *[28]. The default limits for level 3 and 4 were primarily based on results reported by Liebich *et al. *[29] and Rhee *et al. *[31]. Level 3 probe sequences have low cross-hybridization potential and at level 4 the cross-hybridization potential is expected to be significant. The cross-hybridization level assignment described will be considered later in steps 5 and 6 for selecting optimal probes. In summary, at every consecutive selection window or gap all probe sequences are sorted in the order of least potential cross-hybridization first and assigned to the corresponding level. Probe sequences from the most stringent level are collected for further evaluation while less specific probes are excluded.

### Step 4: Estimation of probe self-annealing potential

Stable secondary structure imposed by the probe sequence itself can affect the binding efficiency with the intended target sequence. The self-annealing potential of a probe can be calculated as the minimum free energy folding of the probe sequence. In this step each probe sequence is subjected to a minimum free energy calculation using the hybrid-ss-min program contained in the UNAFold software developed by Markham and Zuker [33,34]. UNAFold computes the free energy using nearest-neighbor coefficients intended for DNA as the 'unified' parameters defined by SantaLucia [34]. The lower the minimum free energy (i.e., more negative), the more stable the secondary structure of the probe. Thus, a higher free energy value (i.e., positive or less negative) indicates a better probe candidate.

### Step 5: Probe selection at fixed intervals

**Figure 2:**
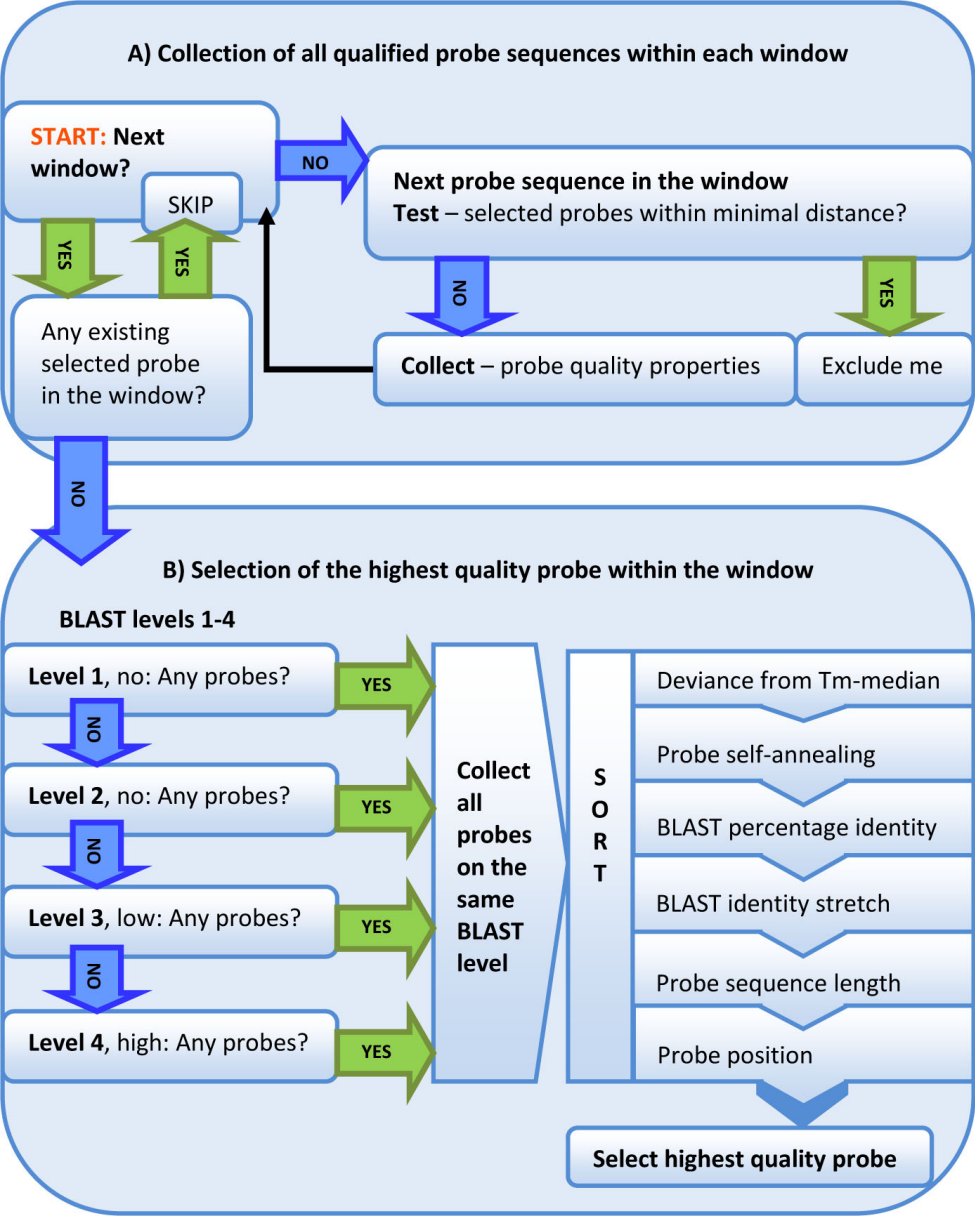
**Schematics of the probe evaluation within selection windows**. **A) Collection of all qualified probe sequences within each window**. All probe properties are collected for every probe sequence that starts within the same selection window. Probes closer than minimal distance to existing selected probes are excluded and windows containing already selected probes due to repeated sequences are omitted. **B) Selection of the highest quality probe within the window**. Screening for the best probe within each selection window starts in the order of cross-hybridization level 1 through 4. Probe sequences of the same level are evaluated based on the following criteria: deviance from median Tm; probe self-annealing; BLAST percentage identity and identity stretch; and, probe length and position.

This step is to select a representative probe candidate within preset nucleotide intervals, referred to as selection windows. From each window, an optimal probe candidate is selected to represent all the probe sequences that start within the same window. The window size (WS) can either be set by users or calculated based on the combined length of all the genomic sequences (GS) and the total number of probe spots available on the microarray (MS):

In the calculation, GS is doubled to include both strands. The reason for truncating the result (i.e., the *floor *function) is because a selected probe of repeated sequence will often represent multiple selection windows. Most genomes contain significant portions of repeated sequences and using the truncated WS as the initial window size will likely result in a number of selected non-redundant probes below MS. If the number of selected probes exceeds MS, the probe selection will be repeated with a one base larger window size (i.e., using the *ceiling *function instead of *floor*). For all probe sequences that start within the same selection window, multiple screening criteria are considered to identify and select the best probe candidate (Figure 2).

To ensure a probe set more evenly distributed across the target genome, a minimal distance (in bases) is kept between any two adjacent selected probes. The minimal distance can either be defined by users or calculated based on the window size. Since the probe selection is performed in a linear manner from the beginning of a genomic sequence, any probe candidate of the next selection window that is too close (i.e., < minimal probe distance) to the previously selected probe is automatically rejected. As repeated probe sequences selected from one window also appear in other windows the script will exclude probes that fall within the minimal probe distance to any existing selected probes elsewhere in the genome. Similarly, if the current window already contains an existing selected probe candidate due to a repeated probe sequence selected from a previous window, no additional probe will be selected.

To select the highest quality probe from all probe sequences occurring in the same window, candidates are grouped based on four levels of cross-hybridization assessed in Step 3. The screening process starts in the order of cross-hybridization level 1 through 4. All probe sequences within the first and most stringent level holding any probes are collected for further evaluation, while the probe sequences not qualifying at this specificity level are excluded. The best probe candidate is then identified in the order of least deviation from median Tm, least probe self-annealing potential, shortest BLAST continuous identity stretch, lowest BLAST percent identity, and longest probe length. If there is more than one best probe candidate (i.e., all criteria of the same value), the first probe encounter will be selected, as this maximizes the distance to the next selection window.

Once a probe is selected, the script will record the genomic coordinates of the probe. For repeated probe sequences multiple positions are recorded. The output generated is a set of probes optimally selected from selection windows tiled along the genomic sequences in both forward and reverse-complement directions.

### Step 6: Additional probe selection from the larger gaps

**Figure 3:**
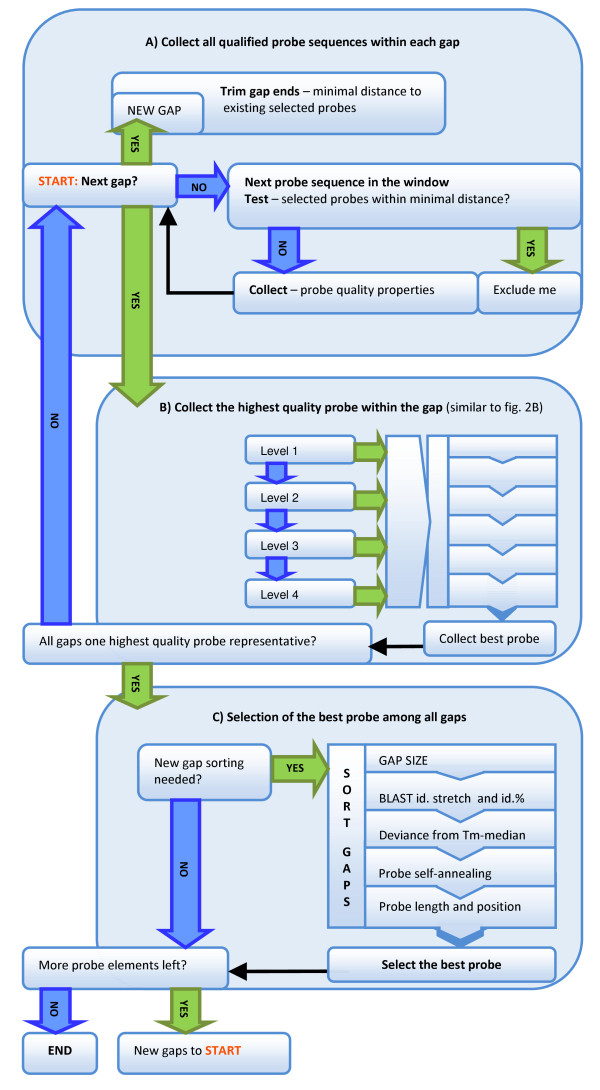
**Schematics of the gap-filling**. **A) Collect all qualified probe sequences within each gap**. This involves removing probe sequences at the gap ends and other repeated probe sequences that appear within minimal distance to already selected probes. **B) Collect the highest quality probe within the gap**. In the first probe screening step, one best probe representative from each gap is collected based on the same ranking criteria used in Figure 2B. **C) Selection of the best probe among all gaps**. The best probes, one highest quality probe from each gap, are ordered by descending gap size and then by the following criteria: BLAST percentage identity and stretch; deviance from median Tm; probe self-annealing; and, probe length and position. The highest quality probe from the largest gap is selected. Every additional probe selected generates two new gaps which are orderly added to the existing gaps. The gap-filling is repeated until all probe spots on the target microarray are used.

The total number of probes selected from fixed sized selection windows is always smaller than the number of probe spots available on the microarray. The remaining spots are filled with additional probe candidates by selecting the highest quality probes from the largest gaps between any two adjacent existing selected probes (Figure 3). The minimal distance between any two adjacent probes is maintained by excluding probe sequences to close to probes that have already been selected. The best probe is then collected from each gap using the same selection criteria as in Step 5. Next, the best probes from all gaps are first ranked by gap size and then by other probe quality criteria (i.e., BLAST continuous identity stretch, BLAST percent identity, deviation from median Tm, probe self-annealing potential, probe length, and probe position). The top ranked probe is finally selected. When a gap is filled with an additional probe, two new gaps are generated. The best probe from each of the new gaps is identified and subjected to the ranking process. The gap-filling is repeated until all probe spots on the microarray are used.

The output of this step, also the final output of the pipeline, is a tiling probe set with exactly the same number of non-redundant probe sequences as there are probe spots on the target microarray.

### Program parameters

The pipeline was implemented with several adjustable parameters both to fit different microarray platforms and to allow for flexibility in the probe set design. A short description of the most important parameters follows:

#### Maximum and minimum probe sequence length

Maximum probe length is the initial desired probe length while providing a shorter minimum probe length allows for isothermal probe design by sequence length adjustment.

#### Total number of probes

The algorithm can design any exact number of probes.

#### BLAST level limits

BLAST limits for defining different levels of expected cross-hybridization is described in the Implementation section (Step 3), using the default level limits. These level limits are user adjustable.

#### Probe selection window size

Default window size is determined by the genome size and by the number of probe spots on the microarray, as described in the Implementation section (Step 5). The pipeline also accepts a user-defined window size.

#### Minimal distance between adjacent probes

Minimal distance between adjacent selected probes is determined by the window size or predefined by the user.

## Results

We have applied the described pipeline to design genomic tiling array probe sets for several microbial genomes that are currently available in the NCBI database [35]. Table 1 shows the statistics of the probe sets designed for various sizes of genomes. The number of probes was preset according to the genome size in order to fit current microarray formats available from Roche NimbleGen, Inc. Default settings were used for all probe set designs. The window size was determined based on the genome size and the number of probe spots on the microarray. Most often the total number of non-redundant probes will be less than the combined number of start positions on both strands. This is because of repeated sequences that are only represented by a single element on the microarray but has more than one position in the genome. Note that for *Candidatus *Carsonella ruddii PV the combined number of start positions is equal to the number of probes, thus none of the selected probes are repeated sequences. These probe sets can be downloaded at our project web site [36]. Additional probe sets for other microbial genomes will be gradually added to the pipeline and sequences made downloadable upon completion.

**Table 1:** Summary statistics of designed probe sets for several microbial genomes of different sizes

Species	*S. cellulosum *'So ce 56'		*P. gingivalis *W83		*Candidatus *C. ruddii PV	
**Genome size**	Large, 13 Mbp		Medium, 2.34 Mbp		Small, 0.16 Mbp	
**Probe number (NimbleGen format)**	1620 K (12*135 K)		385 K		72 K (4*72 K)	
**Window size**	16		12		5	
**Min. distance = smallest gap size**	4		3		2	
**Total non-redundant probes**	1620000		385000		72000	
**Total forward strand starts**	818857		204991		36069	
**Total reverse-compl. strand starts**	818947		205024		35931	

	**Gap size**	**Number**	**Gap size**	**Number**	**Gap size**	**Number**
	
**Probe distribution**	4	123253	3	42959	2	24802
	5	66143	4	22608	3	11222
	6	55731	5	21595	4	20978
	7	54928	6	20832	5	9846
	8	62274	7	21503	6	5152
	9	59254	8	25340		
	10	63218	9	25927		
	11	79015	10	28351		
	12	74532	11	39237		
	13	78092	12	24979		
	14	92854	13	21136		
	15	111273	14	19371		
	16	78459	15	17735		
	17	75716	16	16567		
	18	61562	17	17007		
	19	57317	18	16602		
	20	59414	19	12493		
	21	50445	20	10402		
	22	47169	21	5371		
	23	49842				
	24	42842				
	25	41557				
	26	41786				
	27	32553				
	28	28835				
	29	27753				
	30	21987				

### Program Execution

Due to the comprehensive testing of all the possible probe sequences, the pipeline was implemented on a computer cluster containing multiple nodes and CPU cores. The pipeline was written as a series of multiple Perl scripts for each of the major steps described in the Implementation section. The Perl scripts were executed on a single computer node and when needed the calculations were batched and queued to multiple CPUs in the cluster. Depending on the genome size, a significant amount of time may be required for the BLAST search and the BLAST jobs were submitted to the multi-node multi-core computer cluster using the TORQUE resource manager in combination with the MAUI scheduler downloaded from the Custer Resources web site [37].

### Bench marking

The time limiting step of the pipeline is the millions of BLAST search jobs for estimating the cross-hybridization. Final run time is dependent both on the genome size and on the portion of low complexity sequence. The sizes of the current completed microbial genomes available from NCBI [35] range from ~0.07 Mbp to ~13 Mbp in length. Table 2 shows the pipeline run time for microbial genomes of different sizes.

**Table 2:** Pipeline run time for different probe design sets

**Genomes**	***Cand.*C. ruddii PV**	***B. garinii *Pbi**	***P. gingivalis*W83**	***E. coli *K12 MG1655**	***S. cellulosum *'So ce 56'**
	
Genome size	~0.16 Mbp	~1.22 Mbp	~2.34 Mbp	~4.6 Mbp	~13 Mbp
Characteristic	Small	Multiple	Oral pathogen	*E. coli*	Large
Probe number (NG format)	72 K (4*72 K)	192.5 K (385 K/2)	385 K	770 K (2*385 K)	1620 K (12*135 K)
Probe size	50	50	50	50	50
Min. probe size	45	45	45	45	48
**Run time **					
Step 1	<1 min	<2 min	2 min	6 min	16 min
Step 2	<1 min	8 min	16 min	31 min	1 h 3 min
Step 3 (blastn, -W 7)*	12 min	1 h 43 min	2 h 57 min	6 h 37 min	76 h
Step 4 (hybrid-ss-min)*	6 min	43 min	69 min	2 h 25 min	8 h
Step 5,6	<1 min	7 min	6 min	11 min	31 min
**Total run time**	**~15 min**	**~2 h**	**~3 h 21 min**	**~7 h 25 min**	**~77 h 50 min**

*Run in parallel, only most time consuming step is included in the total run time. Word size 7 is used for blastn.

### Availability

The computational algorithms described were not intended to be implemented as standalone software because of extensive compilation and comprehensive testing for all the possible probe sequences of the target genome. However, we welcome requests from researchers to custom design tilling microarray probe sets. Requests can be sent to the corresponding author or posted via our submission form available at the project web site [36].

## Discussion

To optimize for comprehensive transcriptional mapping of microbial organisms the pipeline described intends to achieve a high probe coverage resolution across the entire genome while maintaining the best possible probe quality. The pipeline ensures an even distribution of probes by maintaining a fixed probe selection window size. An optimal probe sequence is dynamically selected from each window even if all probe candidates within the same window possess poor thermodynamics or strong cross-hybridization potential. Probe position inside the window is less important; hence, the probe sequences selected have an uneven spacing within the limits of the window size. Using a tile path design program, like OligoTiler [12,38] most probes selected will have consistent spacing (i.e., user-defined distance) except when the probe sequence is of low quality, the algorithm will then select a better probe near by. Other dynamic probe selection methods for tiling designs using quality threshold limits tend to leave larger gaps in the low complexity regions, resulting in a less even probe coverage in these areas. While it is true that lower quality probes may distort the resulting hybridization signal intensity, this drawback can be rectified by the use of probe quality-to-signal correction methods like DNA reference arrays [39].

In the pipeline we use minimum free energy of folding for evaluating the probe self-annealing potential. Minimum free energy of folding can also be estimated for the duplex of a probe and its intended or unintended targets. The minimum binding free energy of a probe and its perfectly matched target should be as negative as possible to ensure a stable duplex formation and high hybridization efficiency. Increased signal intensity has been observed with a more negative minimum binding free energy of the perfect match duplex formation [40-42]. On the other hand, the minimum binding free energy of a probe and any non-targets should be as positive as possible (i.e., less negative) to avoid stable duplex formations that may contribute to unspecific signals [43]. It has been reported that the cross-hybridization can be minimized extensively by designing probe sequences with minimum binding free energy between the probe and non-targets less than ca. 50% of the minimum binding free energy between the probe and intended target (i.e., -35 kcal/mol for probe and non-target duplexes to -70 kcal/mol for probe and intended target duplex) [27,29]. Calculation of the exact minimum binding free energy between a probe and a potential non-target, however, requires comparison of all alignments with high identities [44]. Algorithms estimating target and non-target properties, such as target secondary structure [45] and duplex formations [44], generally involve multiple calculations for each probe sequence. These calculations are complex due to the multiple potential non-targets to each probe sequence and because of unpredictable target and non-target fragment lengths. Due to these complications, minimum binding free energy is not evaluated in the current pipeline. However, melting temperature is correlated to annealing temperature and is an alternative estimate for the stability of the duplex formation. This probe design pipeline evaluates and uses melting temperature as an alternative approach to achieve isothermality for selected probes.

As discussed above target lengths may vary and precise calculations for minimum energy of folding are challenging. Compared to the probe sequence, longer target molecules tend to have stronger intra-molecular structures inhibiting the hybridization of the probe to its target. It has been reported that fragmentation of target molecules improves the probe-target binding efficiency by destabilizing the intra-molecular secondary structure [46]. Similarly shorter probe sequences are less likely to form stable secondary structures than longer ones. Although secondary structures may rarely affect the hybridization signal intensity [47] strong self-annealing can still render a probe non-functioning [48]. A more recent study by Wei *et al. *[22] comparing multiple properties of oligonucleotide probes also reported secondary structure to be of high importance to hybridization signal intensity. Methods based on UNAFold (/Mfold) for estimation of probe self-annealing have been used in several probe design applications [49-51] and were included in the pipeline to avoid less functional probes.

For the cross-hybridization assessment, probe candidates within the same window or gap were sorted into four levels based on specificity (i.e., BLAST results). Selecting the best probe from ranked cross-hybridization levels ensures that the probe sequences with no or the least cross-hybridization will always be considered first albeit less ideal thermodynamic properties. Evaluation of other criteria for probes within the same level and thus with similar cross-hybridization potentials is also more meaningful. This tiered specificity categorization is more robust than a straightforward multiple sorting method for identifying a better probe candidate.

Unique to other tile design applications we added an iterative second selection step, selecting probes from the larger gaps generated between existing selected probes. Adjusting the window size in the first selection step will emphasize either window or gap selection. A larger initial window size results in more and larger gaps for the gap-filling step. Although larger window size provides more sequences for probe selection, hence better probe quality, the trade-off is a less even probe coverage on the genome. Consequently, the adjustment of the window size for better probe quality must be balanced with the probe coverage. The pipeline also provides the opportunity to preset the minimal allowed distance to neighboring probes. The minimal distance directly determines the smallest spacing between selected probes and will also influence the number of available probe sequences to choose from in each window and gap. Our intention by combining the two selection steps together with optional settings of both minimal distance and window size, was to allow for a more flexible probe design.

## Conclusions

We have described a working computational pipeline consisting of multiple algorithms, specifically developed for designing tiling array probe sets to study microbial transcriptomes. The pipeline evaluates several probe properties in multiple steps and dynamically selects the best probe candidates from fixed size selection windows that are tiled along the target genome in both forward and reverse-complement directions. The pipeline is capable of designing a probe set with an exact number of non-redundant probe sequences to utilize every probe spot that can be printed on a microarray. This is achieved in two iterations of probe selection - sequential and gap-filling. Due to the high computational power needed for the extensive calculation of probe qualities, the pipeline was not designed to be executed on personal computers. Free services for academic researchers are available upon request and custom tiling probe sets can be designed with the computer cluster in our laboratory.

We have successfully designed genomic tiling arrays using this pipeline, e.g. to study the transcriptome of the oral pathogen *Porphyromonas gingivalis*. Preliminary transcriptome data can be viewed at our project web site [36].

## Availability and requirements

**Project name: **Dynamic probe selection for studying microbial transcriptome with high-density genomic tiling microarrays

**Project home page: **http://bioinformatics.forsyth.org/mtd

**Operating system: **Web-based application based on Linux OS

**Programming language: **PERL

**Other requirements: **None

**License: **GNU GPL

**Any restrictions to use by non-academics: **Fee-based service

## Authors' contributions

HH and TC contributed equally to all parts of this project. Both authors have read and approved the final manuscript.
